# Supplementary material for: Autoinflammatory patients with Golgi-trapped CDC42 exhibit intracellular trafficking defects leading to STING hyperactivation and ER stress
Source: Nat Commun. 2024 Nov 16;15:9940. doi: 10.1038/s41467-024-54294-y (PMC11569173; doi:10.1038/s41467-024-54294-y)
Supplement: Supplementary file 1 — Supplementary Information [file 41467_2024_54294_MOESM1_ESM.pdf]

Supplementary information of the manuscript by Iannuzzo *et al.* entitled "Autoinflammatory patients with Golgi-trapped CDC42 exhibit intracellular trafficking defects leading to STING hyperactivation and ER stress"

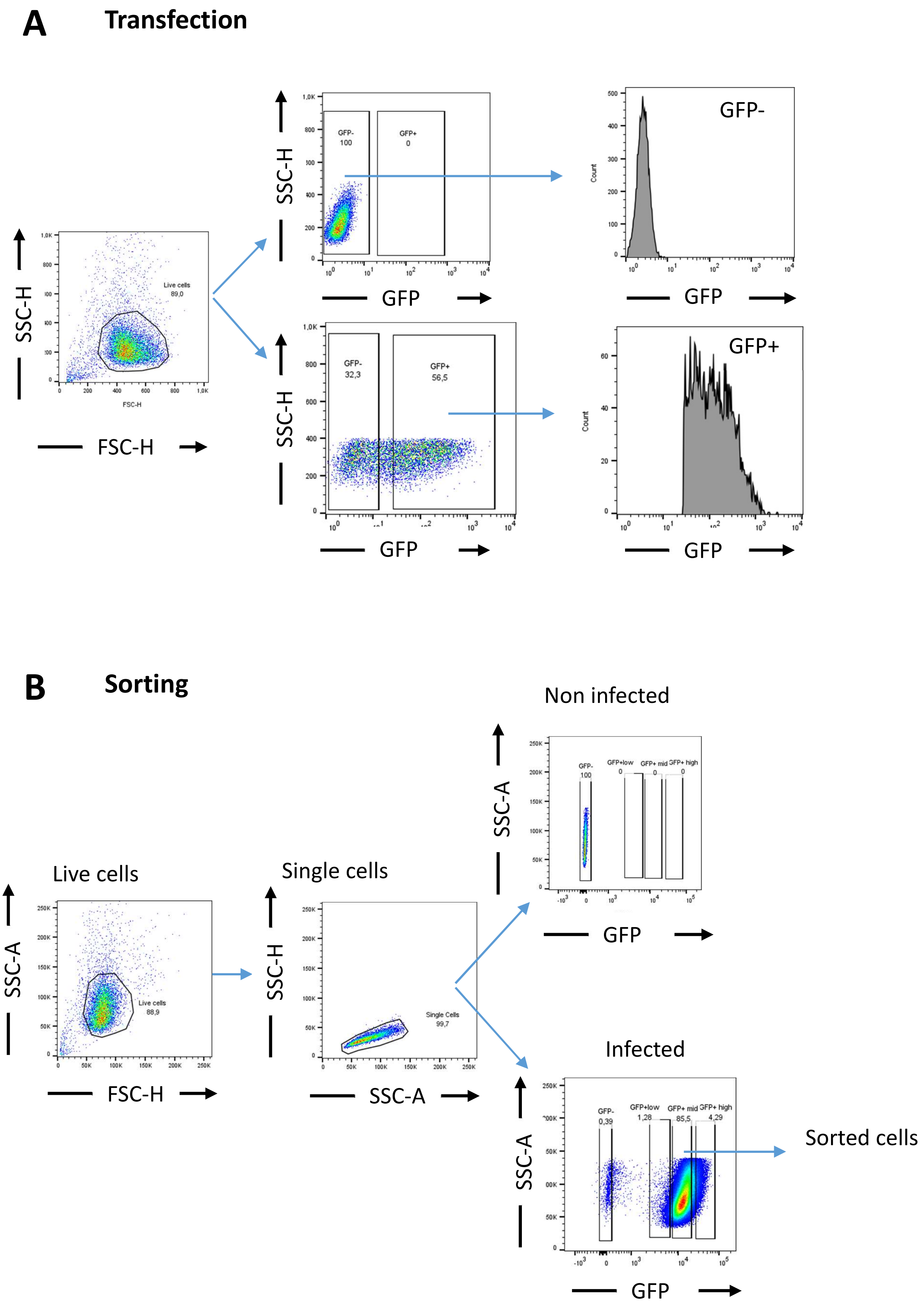
A
Transfection
SSC-H
FSC-H
Live cells
89,0
GFP-
100
GFP+
0
GFP
Count
GFP-
GFP-
32,3
GFP+
56,5
GFP+
B
Sorting
Live cells
SSC-A
FSC-H
Live cells
88,9
Single cells
SSC-H
SSC-A
Single Cells
99,7
Non infected
GFP-
100
GFP+low
GFP+ mid
GFP+ high
Infected
GFP-
0,39
GFP+low
1,28
GFP+ mid
85,5
GFP+ high
4,29
Sorted cells

Supplementary Figure 1: Flow cytometry gating strategy. **A,** GFP expression levels and gating strategy in THP-1 cells transiently expressing GFP-tagged CDC42 variants. **B,** Sorting strategy used in THP-1 cells infected with the different CDC42 variants.

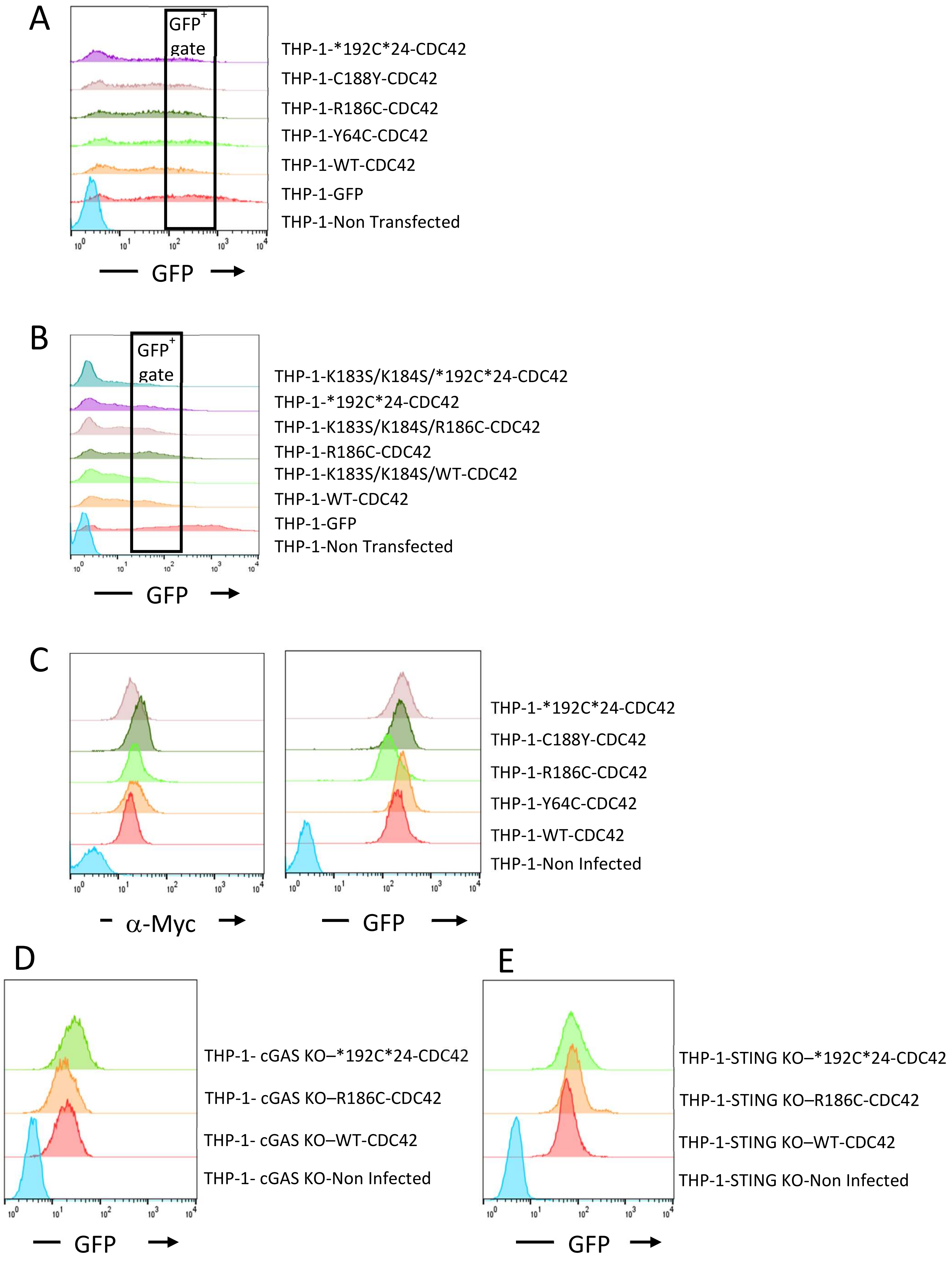
A
GFP+ gate
THP-1-*192C*24-CDC42
THP-1-C188Y-CDC42
THP-1-R186C-CDC42
THP-1-Y64C-CDC42
THP-1-WT-CDC42
THP-1-GFP
THP-1-Non Transfected
GFP
B
GFP+ gate
THP-1-K183S/K184S/*192C*24-CDC42
THP-1-*192C*24-CDC42
THP-1-K183S/K184S/R186C-CDC42
THP-1-R186C-CDC42
THP-1-K183S/K184S/WT-CDC42
THP-1-WT-CDC42
THP-1-GFP
THP-1-Non Transfected
GFP
C
THP-1-*192C*24-CDC42
THP-1-C188Y-CDC42
THP-1-R186C-CDC42
THP-1-Y64C-CDC42
THP-1-WT-CDC42
THP-1-Non Infected
α-Myc
GFP
D
THP-1- cGAS KO–*192C*24-CDC42
THP-1- cGAS KO–R186C-CDC42
THP-1- cGAS KO–WT-CDC42
THP-1- cGAS KO-Non Infected
GFP
E
THP-1-STING KO–*192C*24-CDC42
THP-1-STING KO–R186C-CDC42
THP-1-STING KO–WT-CDC42
THP-1-STING KO-Non Infected
GFP

Supplementary Figure 2: Flow cytometry histograms of transiently transfected or transduced THP-1 cells. **A, B:** GFP$^+$ gate in THP-1 cells transiently transfected with GFP alone or with GFP-tagged WT or variants CDC42. Parental (**C**), cGAS KO (**D**) and STING KO (**E**) THP-1 cells transduced with a bicistronic lentiviral vector that encodes for both Myc-tagged CDC42 variants and GFP.

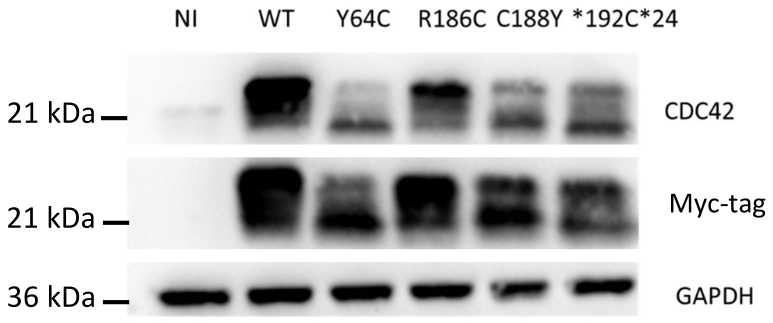

Supplementary Figure 3: CDC42 expression in THP-1 cells transduced with CDC42 variants. Western blot analysis of Myc-tag and CDC42 expression in THP-1 cells transduced with WT or variants CDC42. The GAPDH immunoblot is shown as a loading control. NI: non-infected. Source data are provided as a Source Data file.

PATIENTS		P1	P2	P3	P4	P5	P6	P7
CDC42 mutation		R186C/WT	R186C/WT	R186C/WT	R186C/WT	*192C*24/WT	*192C*24/WT	Y64C/WT
References		5	5	8	10	10	9	16
	First manifestations	Neonatal	Neonatal	Neonatal	Neonatal	Neonatal	Neonatal	Neonatal
	Gender	Female	Male	Male	Female	Male	Female	Female
	Outcome and status	Alive, 10 yr	Dead, 7 mo	Dead, 4.5mo	Alive,10.5yr	Alive, 16 yr	Dead, 55 yr	Dead, 26yr
	Recurrent fever	+	+	+	+	+	-	-
	Failure to thrive	+	+	-	+	+	+	+
	Skin rash	+	+	+	+	+	-	-
	Hepatosplenomegaly	+	+	+	+	+	+	+
	Dysmorphic features	-	-	-	-	+	+	+
	CNS inflammatory disease	+	-	-	+	+	-	-
	HLH episode	+ (4 episodes)	+ (1 fatal episode)	+	+	-	-	-
Hematology	Pancytopenia	+	+	+	+	+	-	+
	Elevated inflammatory markers	+	+	+	+	+	-	+
	BM dysplasia	+	+	-	Not tested	Not tested	-	+
Serum / plasma avaibility		onset; 1; 2; 3; 4	onset; 1	onset; 1	1; 2	1; 2	1; 2	-
Treatment		-; Steroids Anakinra Cyclosporine; -	-; Steroids Anakinra	-; Etanercept	Anakinra; Canakinumab Steroids	Anakinra	Steroids and IgG maintenance therapy	Steroids
HSCT		-; +	-	-	-	-	-	-

Supplementary Table 1: Clinical features of the seven CDC42 patients studied here.

CNS: Central Nervous System. HSCT: Hematopoietic Stem Cell Transplantation.

cDNA qPCR	Gene	Forward Primer (5'-3')	Reverse Primer (5'-3')
	IFI27	CTCTGCCGTAGTTTTGCCCC	ACACTAACCTCCTCAACACCGG
	IFI44L	AGTTTAATTCCCGTAAACCA	CAACCGTTTTCACTTCGTTC
	IFIT1	ATGAGTACAAATGGTGATGA	CAGAACCTAGCTAACTTAA
	ISG15	GGTGGACAAATGCGACGAACCTC	ACTCGCCCGACCCCCACAC
	RSAD2	GCGTCAACTATCACTTCACTCG	CAGGTATTCTCCCCGGTCT
	SIGLEC1	ACAACTTCCGCTTCGAGATCAGT	AGAGCTCCCGTGTCTCCACCT
	HSPA5	CAAGCAACCAAAGACGCTGGA	CTCCACTGTTTGGCTATGGACA
	DDIT3	AGAGGAAGAATCAAAAATCT	AGGTCTAAGGTCAGTCTCGA
	ATF4	GTTCTCCAGCGACAAGGCTA	CTACGGGACAACCCATATCT
	GAPDH	GTCTCCTCTGACTTCAACAGCG	AACCGATGTCGTTGCACCA

Supplementary Table 2: Identity and sequences of the qPCR primers used in this study.

Primary Antibody	Host species	Source	Catalog Reference	Lot Number
Anti-PC-1	Rabbit	Rockland	600-401-103s	XG3633015
Anti-GM130	Mouse	BD Biosciences	610822	2025007, 54028
Anti-SERCA2 ATPase	Goat	Abcam	ab219173	
Anti-Giantin	Rabbit	Abcam	ab80864	1065654-1
Anti-STING-TMEM173	Mouse	R&D System	#723505	CFWR0420041
Anti-P-STAT1 (Tyr 701)	Rabbit	Cell Signaling	CST7649	
Anti-P-IRF3 (Ser 396)	Rabbit	Thermo Fisher Scientific	720012	2352344
Anti-GRP78 BiP	Rabbit	Abcam	ab21685	
Phalloidin-AF488		Thermo Fisher Scientific	A12379	1917950
Hoechst		Thermo Fisher Scientific	H21486	
Donkey anti Mouse IgG- Alexa 647		Invitrogen	A32787	Y5378039
Donkey anti Rabbit IgG- Alexa 647		Invitrogen	A31573	2674379
Donkey anti Rabbit IgG- Alexa 568		invitrogen	A10042	2433862
Donkey anti Goat IgG- Alexa 488		Invitrogen	A11055	2465077
Donkey anti Rabbit IgG- Alexa 488		Invitrogen	A21206	2330673

Anti-GAPDH	Mouse	Santa Cruz Biotechnology	sc-47724	H2521
Anti-Myc-tag (9B11)	Mouse	Cell Signaling	2276S	19
Anti-CDC42	Rabbit	Cell Signaling	2462S	4
HRP-conjugated anti- rabbit IgG	Goat	Jackson immunoResearch	#111-035-144	
HRP-conjugated anti- mouse IgG	Goat	Jackson immunoResearch	#115-035-146	131600

Supplementary Table 3: List of staining reagents used.

Supplemental material

CLINICAL CHARACTERIZATION OF CDC42 PATIENTS

Patient 1

P1, carrying the R186C CDC42 mutation described in[5], was a girl born from healthy unrelated parents with no history of genetic disease. At birth, she presented with high fever, a diffuse erythematous skin rash, hepatosplenomegaly and failure to thrive. Blood analysis showed elevated inflammatory markers. A bone marrow (BM) biopsy, performed at the onset of the disease, revealed fibrosis with dyshematopoiesis. Treatment with glucocorticoids and daily therapy with anakinra improved the fever and rash but had no effect on the cytopenia. Therefore, treatment with G-CSF was started with partial response. Following tapering and/or discontinuation of glucocorticoids, a recurrence of inflammatory symptoms was observed. At 11 months, she had several episodes of intestinal bleeding. At 2 years and 6 months, she presented with three episodes of generalized seizures. Cerebral MRI was suggestive for central nervous system (CNS) inflammation in the absence of infection. The episodes were treated with high doses of glucocorticoids with good response. From the age of 5 years old, she developed four episodes of Hemophagocytic Lymphohistiocytosis (HLH). All episodes, except the final one, were resolved with treatment with high dose glucocorticoids and cyclosporine-A. During the last episode, she did not respond to high-dose glucocorticoids and IL-1 inhibition, and finally required surgical resection and consequent ileocolostomy due to massive intestinal ischemia and necrosis. However, the addition of emapalumab, an anti–IFN-γ antibody, induced a rapid resolution of the episode.

Serial measurements of the IFNα-2a concentration in the plasma of this patient was performed during the course of the disease. She displayed a high level of IFNα-2a at the onset of disease, which decreased under therapy with glucocorticoids and anakinra (Figure 7), as demonstrated

in time point 1. At time point 2, P1 had an adenovirus and rhinovirus infection. Despite continuous treatment with glucocorticoids, anakinra and cyclosporine, the IFNα levels remained high. The patient finally underwent a Hematopoietic Stem Cell Transplantation (HSCT), which restored normal IFNα-2a concentration (point 4).

Patient 2

P2, who carried the R186C CDC42 variant and described in[5], was a male born from healthy unrelated parents. He presented at birth with persistent fever, skin rash, hepatosplenomegaly, failure to thrive, increase in inflammatory markers and pancytopenia, requiring red blood cell and platelet transfusions. Dyshematopoiesis and some lymphohistiocytic aggregates, without significant hemophagocytosis, were discovered by BM biopsy. The disease course was characterized by a persistent inflammatory state despite treatment with glucocorticoids, high doses of immunoglobulins and cyclosporine-A. Suspecting an autoinflammatory condition, treatment with anakinra was also started with only partial improvement of the clinical and laboratory parameters. At 7 months, he developed a severe HLH with multiorgan failure and rapidly progressed to death.

IFNα levels were measured at different time points in the plasma during the course of the disease. At the onset of disease, the IFNα-2a concentration was very high (Figure 7) and decreased after the addition of Anakinra, as indicated in time point 1.

Patient 3

P3, carrying the R186C CDC42 mutation and described in[8], was the first male child born from non-consanguineous parents. Neonatally, he presented with fever, erythema, hepatosplenomegaly and pancytopenia. He did not demonstrate dysmorphic features.

Treatment with high-dose immunoglobulins was only partially effective and addition of glucocorticoids was necessary to stabilize the patient's condition. Tapering of the glucocorticoids induced a recurrence of fever and erythema. Since re-escalation of prednisolone could not suppress inflammation, Etanercept was added to the treatment. The inflammation could not be controlled and the patient died at 4.5 months due to overwhelming inflammation.

IFNα-2a levels were measured at 2 different time points in the patient's serum. The C-reactive protein (CRP) level was low at the first time point (1.7 mg/L) and elevated at the second time point (15.6 mg/L). At the onset of disease, the IFNα-2a concentration was high and decreased after the initiation of the treatment with Etanercept, as indicated in time point 1 (Figure 7).

Patient 4

P4 who carries the R186C CDC42 variant was described in[10]. She is a girl who presented at birth with a rash, grade II brain hemorrhage, hepatosplenomegaly and thrombocytopenia. She had frequent febrile episodes and chronic cytopenia. Her anemia and thrombocytopenia were transfusion-dependent. The parents were non-consanguineous. There was a poor response to glucocorticosteroids and rituximab. At the age of 5 months, she started on anakinra with improvement of the rash and fever. Although the dose of Anakinra was gradually augmented, there was an amelioration of the anemia, thrombocytopenia, hyperferritinemia and systemic inflammation but no complete resolution. Upon switch of Anakinra to Canakinumab, an improvement of the anemia and thrombocytopenia was noticed and the white blood cell count normalized. Yet she still suffered from recurrent episodes of anterior cervical lymphadenitis and chronic hepatosplenomegaly. The patient is currently 10.5 years old and receiving treatment with canakinumab (Figure 7).

Patient 5

P5, carrying the *192C*24 mutation in CDC42, is a 15-year-old male patient described in[10]. His parents are non-consanguineous. He presented neonatally with fever, erythematous rash, hepatosplenomegaly, anemia and thrombocytopenia. At 7 weeks, he had an intracranial hemorrhage secondary to the severe thrombocytopenia. Until the age of 8 months, transfusion-dependent anemia and thrombocytopenia, hepatosplenomegaly, recurrent fever and rash persisted. Glucocorticoids and anakinra were started at the age of 8 months, based upon a clinical suspicion of NOMID, which resulted in a resolution of the systemic inflammation and cytopenias. He suffered from respiratory syncytial virus–induced pneumonia, septic arthritis, and cervical lymphadenitis. Recurrent infections resolved upon discontinuation of the steroids. Currently, the patient's systemic inflammation characterized by fever, rash, anemia, and thrombocytopenia has resolved and there is an absence of hepatosplenomegaly. The patient is currently 15 years-old and receiving treatment with canakinumab (Figure 7).

Patient 6

P6, who carried the *192C*24 CDC42 mutation, was described in[9]. She presented in early childhood with a short stature resembling achondroplasia, subtle dysmorphic features, hepatosplenomegaly, subglottic stenosis, scleritis, and inflammation of both auricles. During childhood, she suffered from recurrent infections including multiple pneumonias and an episode of viral meningitis. Blood analysis revealed elevated inflammatory markers, mild anemia, slightly elevated liver enzymes and an isolated IgM deficiency. Hypercellularity with trilineage hematopoiesis interpreted as reactive changes was discovered by bone marrow biopsy. She suffered from several pneumonias which necessitated hospitalization and intravenous antibiotic therapy. Due to the clinical suspicion of relapsing polychrondritis and an underlying primary immunodeficiency, she was treated with glucocorticosteroids and IgG maintenance therapy. When she was 44, a partial jejunum resection was performed after she

suffered from acute abdominal pain due to intestinal necrotizing vasculitis. At age 55, she was admitted with acute abdominal pain and was diagnosed with a paralytic ileus. During admission, her clinical status rapidly declined and eventually she succumbed due to hemodynamic instability and pulmonary hypertension (Figure 7).

Patient 7

P7, carrying the Y64C CDC42 variant and described in[16], was born from non-consanguineous parents. She was small for gestational age, microcephalic, hypotonic and mildly dysmorphic. She manifested psychomotor developmental delay, feeding difficulties and growth retardation. She had microretrognathia, hypertelorism with incomplete eyelid closure, depressed nasal bridge, strabismus, severe astigmatism, arachnodactyly, and sensorineural hearing loss. She developed scoliosis, pes planus, camptodactyly, fingernail exostosis, hepatosplenomegaly with multiple spleen and kidney hyperechogenic lesions (which were not biopsied), liver hemangiomas, and a thoracic-abdominal aortic aneurysm. She suffered recurrent lower respiratory tract infections since childhood with a diagnosis of bronchiectasis at age 15 years despite antibiotic treatment and physiotherapy. Laboratory evaluations at that time showed profound lymphopenia, especially of B cells, neutropenia, anemia, and thrombocytopenia. A bone marrow biopsy at age 16 showed dysmegakaryopoiesis but otherwise normocellular marrow with normal differentiation. She had hypogammaglobulinemia in the first year of life with IgA and IgM deficiency, but only IgA deficiency persisted later in life. At age 25 years, she experienced weight loss (> 10%), increased coughing, fever, and elevated inflammatory markers. C-reactive protein (CRP) and erythrocyte sedimentation rate (ESR) increased. HLH could not be confirmed. The patient's state did not improve despite broad antifungal and antibiotic treatment, but responded to steroids. Bone marrow biopsy showed myeloid hypercellularity, erythroid hypoplasia, and fibrosis grade 2/3 according to the WHO classification of myeloid neoplasm, suggestive of primary myelofibrosis. As part of the

hematology workup, a NGS panel (Illumina platform) on peripheral blood was performed to screen for driver variants in *JAK2*, *CALR*, and *MPL*, as well as for non-driver variants in a large set of genes. No somatic or germline variants could be identified. At age 26 years, she was admitted with cachexia, dyspnea, and hypoxemia. A chest CT scan showed bilateral ground glass opacities, honeycombing, and a crazy paving pattern with cyst formation. Bronchus' aspirate revealed MRSA and HSV-1 for which she was treated accordingly. Ventilatory support was escalated to extracorporeal membrane oxygenation (ECMO) from D+7. Suspecting an inflammatory component, she was started on 80 mg methylprednisolone per day on D+11, after which she could be weaned from ECMO. Upon tapering of steroids, lung infiltrates flared—a pattern seen twice, after which glucocorticoid therapy was maintained from D+41. During this course, she had several episodes of pulmonary hemorrhages. CT angiography showed several hypertrophic bronchial arteriae which were embolized. At D+41 after admission, *Stenotrophomonas maltophilia* was cultured from the sputum. Ultimately, the patient succumbed to shock associated with *Stenotrophomonas maltophilia* bacteremia at D+68 after admission.
